# High-resolution detection of quantitative trait loci for seven important yield-related traits in wheat (*Triticum aestivum* L.) using a high-density SLAF-seq genetic map

**DOI:** 10.1186/s12863-022-01050-0

**Published:** 2022-05-13

**Authors:** Tao Li, Qiao Li, Jinhui Wang, Zhao Yang, Yanyan Tang, Yan Su, Juanyu Zhang, Xvebing Qiu, Xi Pu, Zhifen Pan, Haili Zhang, Junjun Liang, Zehou Liu, Jun Li, Wuyun Yan, Maoqun Yu, Hai Long, Yuming Wei, Guangbing Deng

**Affiliations:** 1 Chengdu Institute of Biology, Chinese Academy of Sciences, Chengdu, 610041 China; 2 Triticeae Research Institute, Sichuan Agricultural University, Chengdu, 611130 China; 3 State Key Laboratory of Crop Gene Exploration and Utilization in Southwest China, Chengdu, 611130 China; 4 Crop Research Institute, Sichuan Academy of Agricultural Sciences, Chengdu, 610066 Sichuan China

**Keywords:** Wheat, Yield, Yield-related traits, Specific-locus amplified fragment (SLAF), Linkage analysis

## Abstract

**Background:**

Yield-related traits including thousand grain weight (TGW), grain number per spike (GNS), grain width (GW), grain length (GL), plant height (PH), spike length (SL), and spikelet number per spike (SNS) are greatly associated with grain yield of wheat (*Triticum aestivum* L.). To detect quantitative trait loci (QTL) associated with them, 193 recombinant inbred lines derived from two elite winter wheat varieties Chuanmai42 and Chuanmai39 were employed to perform QTL mapping in six/eight environments.

**Results:**

A total of 30 QTLs on chromosomes 1A, 1B, 1D, 2A, 2B, 2D, 3A, 4A, 5A, 5B, 6A, 6D, 7A, 7B and 7D were identified. Among them, six major QTLs *QTgw.cib-6A.1*, *QTgw.cib-6A.2*, *QGw.cib-6A*, *QGl.cib-3A*, *QGl.cib-6A*, and *QSl.cib-2D* explaining 5.96-23.75% of the phenotypic variance were detected in multi-environments and showed strong and stable effects on corresponding traits. Three QTL clusters on chromosomes 2D and 6A containing 10 QTLs were also detected, which showed significant pleiotropic effects on multiple traits. Additionally, three Kompetitive Allele Specific PCR (KASP) markers linked with five of these major QTLs were developed. Candidate genes of *QTgw.cib-6A.1/QGl.cib-6A* and *QGl.cib-3A* were analyzed based on the spatiotemporal expression patterns, gene annotation, and orthologous search.

**Conclusions:**

Six major QTLs for TGW, GL, GW and SL were detected. Three KASP markers linked with five of these major QTLs were developed. These QTLs and KASP markers will be useful for elucidating the genetic architecture of grain yield and developing new wheat varieties with high and stable yield in wheat.

**Supplementary Information:**

The online version contains supplementary material available at 10.1186/s12863-022-01050-0.

## Background

Common wheat (*Triticum aestivum* L.) is one of the three major crops worldwide and provides approximately 30% of global grain production and 20% of the calories consumed for humans [1]. Due to ongoing decrease of the global arable cultivated land area and increase of the population, the current rate of wheat yield increase will be insufficient to meet the future demand. Thus, breeding of high-yield wheat varieties to ensure future global food and nutrition security is an important target of the modern wheat breeding programs [2].

Wheat yield is a complex quantitative trait controlled by multiple genes and significantly influenced by interacting genetic and environmental factors [3, 4]. By contrast, yield components including thousand grain weight (TGW), grain number per spike (GNS), grain width (GW), grain length (GL), plant height (PH), spike length (SL) and spikelet number per spike (SNS) typically show higher heritability than that of the yield [5–7]. Therefore, targeting these traits and identifying the related genes or quantitative trait loci (QTL) is an important approach to improve grain yield potential in wheat.

The molecular cloning of genes associated with wheat yield is difficult owing to wheat’s huge and complicated genome. To date, only a few genes associated with grain yield have been cloned in wheat. For example, the application of semi-dwarfing genes *Rht-B1b* and *Rht-D1b* not only effectively improve the lodging resistance but also improve the harvest index, resulting in increasing yield since the 1970s [8–10]. The vernalization insensitive alleles of *Vrn-1* (*Vrn-A1*, *Vrn-B1*, and *Vrn-D1*) shorten both the vegetative and the reproductive stages and have considerable impact on spike morphological traits [11, 12]. The grain-shape gene *TasgD1* encoding a Ser/Thr protein kinase glycogen synthase kinase3 and independently control semispherical grain trait [13]. A jasmonic acid synthetic gene keto-acyl thiolase 2B was cloned in a TGW mutant, showing significant effects on TGW and GW [14]. Additionally, homologous cloning is an effective approach to characterize gene in wheat. As of today more than 20 genes related to yield have been isolated through homologous cloning approach, including *WFZP*, *WAPO1*, *TaGW7*, *TaGW2*, *TaCKX6-D1*, *TaTGW6*, *TaGASR7*, *TaGL3* and *TaGS-D1* et al [15–23].

Quantitative trait loci (QTL) mapping provides an effective approach to dissect the genetic architecture of complex quantitative traits. Over the past decades, numerous QTLs associated with yield or yield-related traits have been identified on all wheat chromosomes [3, 4, 11, 24–30]. For example, *Rht8* located on chromosome 2DS was closely linked with marker *xfdc53* and reduced plant height by 10% [31]; *Rht25* on wheat chromosome arm 6AS showed pleiotropic effects on coleoptile length, heading date, SL, SNS and grain weight [32]. Two major QTLs for grain size and weight were detected on chromosome 4B, which together explained 46.3% of the phenotypic variance [33, 34]. Five stable QTLs for PH, SL and HD on chromosomes 1A, 2A, 2D and 6A were detected in an introgression line population [35]. Twelve major genomic regions with stable QTL controlling yield-related traits were detected on chromosomes 1B, 2A, 2B, 2D, 3A, 4A, 4B, 4D, 5A, 6A, and 7A [1]. However, among these QTLs reported previously, few of them were stably detected in multi–environments, which greatly restrict their potential utilization in marker-assisted selection (MAS) in breeding programs.

With the development of high-throughput sequencing technology, Single nucleotide polymorphisms (SNP) markers have been widely applied to construct high-density genetic maps for QTL mapping, due to their extensive and intensive distribution throughout genomes in many crop s[3, 36–38]. Specific-locus amplified fragment sequencing (SLAF-seq) was developed for economic and efficient high-throughput SNP discovery through restriction-site associated DNA tag sequencing (RAD-seq), which can provide abundant InDel and SNP markers to construct high-density genetic map [39–41].

In the present study, a high-resolution genetic map was constructed in a recombinant inbred line (RIL) population derived from two elite winter wheat varieties Chuanmai42 (CM42) and Chuanmai39 (CM39) based on SLAF-seq (Table S1, S2) [42]. Seven traits including TGW, GW, GL, PH, GNS, SL and SNS were assessed in multi-environments to detect potential major and stable QTL, which will lay out a foundation for further study on fine mapping and cloning of the underlying key genes for wheat yield.

## Results

### Phenotypic variation

**Fig. 1:**
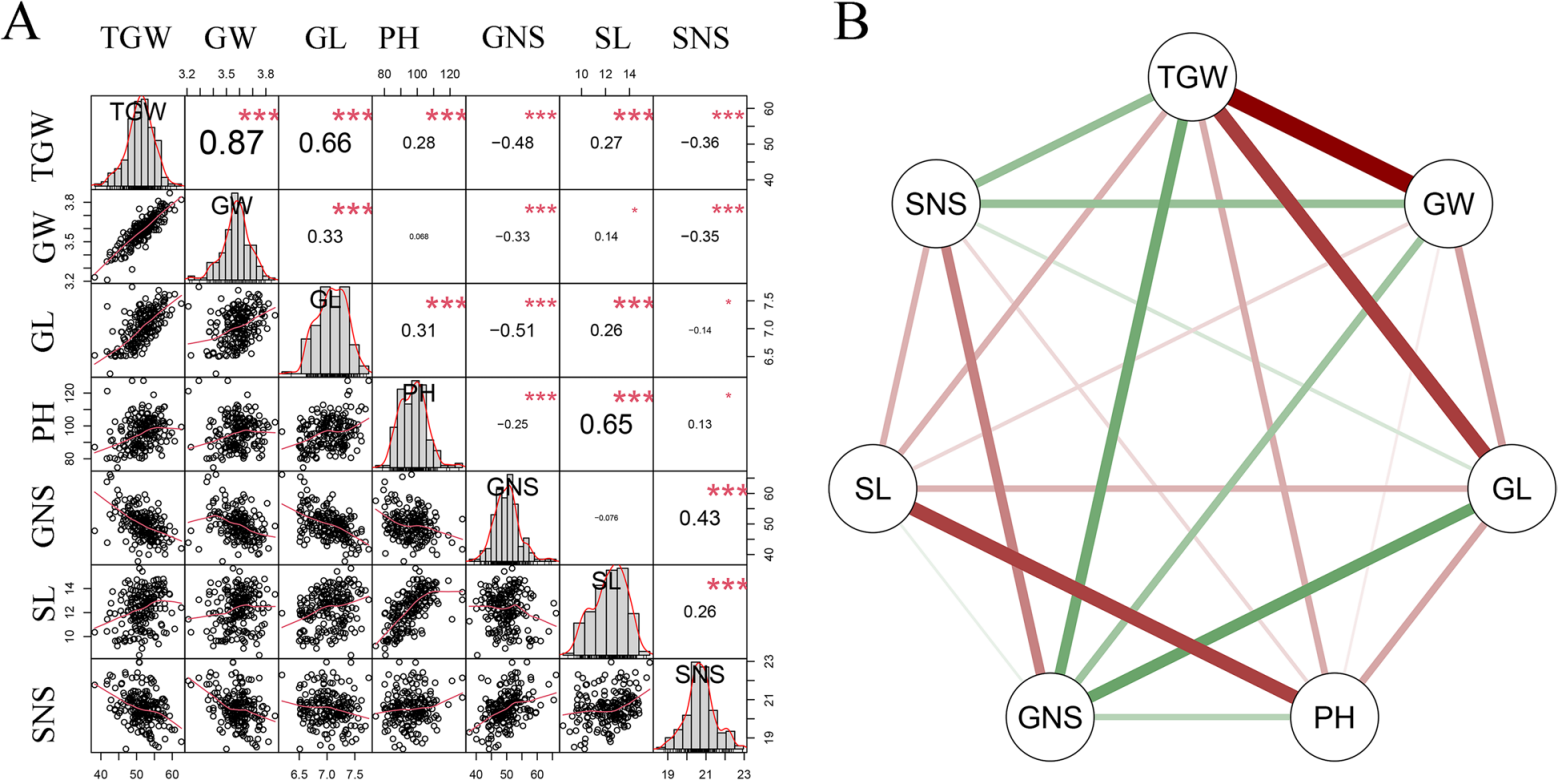
Phenotypic performances, distribution, and correlation coefficients of thousand grain weight (TGW), grain number per spike (GNS), grain width (GW), grain length (GL), plant height (PH), spike length (SL) and spikelet number per spike (SNS) in the CM42×CM39 RIL lines based on the BLUP datasets (**A**). **B** Visualization of correlations among investigated traits; Red and green lines represent positive and negative correlation, respectively; The line weight represent the size of correlation coefficient; *, ** and *** represent significant at *P* < 0.05, *P* < 0.01 and *P* < 0.001, respectively

The phenotypic analysis showed that CM42 had higher trait values for TGW, GW, GL, GNS, PH and SL than those of CM39 in each of environments and the best linear unbiased prediction (BLUP) datasets (Table 1). In the RIL population, seven yield-related traits showed wide and significant variations in all environments and the BLUP datasets (Table 1). Of them, the TGW ranged from 20.81 to 72.7 gram (g), the GW ranged from 2.6 to 4.21 millimeter (mm), the GL ranged from 5.88 to 8.81 mm, the PH ranged from 65.08 to 148.3 centimeter (cm), the GNS ranged from 24 to 84.6, the SL ranged from 6.65 to 18.17 cm, and the SNS ranged from 15.83 to 27, respectively (Table 1). The BLUP datasets of all traits showed normal distributions in the RIL lines, which suggested polygenic inheritance of these traits (Fig. 1A). Additionally, the TGW, GL, PH, GNS and SL showed high across-environment broad-sense heritability of 0.54, 0.6, 0.91, 0.66 and 0.88, respectively (Table 1). Significant and positive correlations (*P* < 0.01) of the seven yield-related traits among all environments and the BLUP datasets were detected, which suggested that these traits were environmentally stable and mainly controlled by genetic factors (Table S3).

**Table 1:** Phenotypic variation of the seven yield-related traits, including thousand grain weight (TGW), grain number per spike (GNS), grain width (GW), grain length (GL), plant height (PH), spike length (SL) and spikelet number per spike (SNS), for the parents and the CM42×CM39 RIL lines in different environments

Traits	Environments	Parents		The CM42×CM39 RIL lines				
		CM42	CM39	Range	Mean	SD	CV (%)	***H***^***2***^
TGW	2017SHF	54	52.94	38.34-70.88	58.57	5.84	9.98	0.54
	2017SHL	50.64	41.83	20.81-68.14	43.76	9.17	20.95	
	2018SHF	54.79	53.47	40.44-72.7	54.67	5.58	10.2	
	2018SHL	53.06	51.29	37.89-67.33	54.51	5.57	10.22	
	2019SHF	52.4	42.42	32.59-66.54	51.27	5.94	11.59	
	2019SHL	51.05	47.38	23.4-62.74	46.9	6.22	13.27	
	BLUP	52.36	50.44	38.24-62.56	51.65	3.98	7.7	
GW	2017SHF	3.68	3.42	3.19-4.21	3.82	0.16	4.28	0.49
	2017SHL	3.54	3.31	2.6-4.01	3.38	0.29	8.57	
	2018SHF	3.58	3.53	3.19-4.04	3.69	0.16	4.35	
	2018SHL	3.63	3.61	3.15-3.96	3.65	0.15	4.14	
	2019SHF	3.6	3.16	3-3.9	3.5	0.18	5.21	
	2019SHL	3.56	3.49	2.84-3.99	3.49	0.19	5.37	
	BLUP	3.59	3.51	3.21-3.87	3.59	0.11	3.06	
GL	2017SHF	7.73	7.17	6.78-8.81	7.76	0.41	5.26	0.6
	2017SHL	6.95	6.53	5.94-7.89	6.86	0.37	5.39	
	2018SHF	6.87	6.72	5.89-7.92	6.95	0.37	5.3	
	2018SHL	7.64	6.55	5.88-7.81	6.85	0.37	5.45	
	2019SHF	7.32	6.43	6-7.71	6.86	0.33	4.84	
	2019SHL	7.22	6.67	6.03-7.71	6.94	0.36	5.15	
	BLUP	7.27	6.98	6.19-7.75	7.04	0.3	4.26	
PH	2016SHF	90.34	89.5	66.5-120.3	91.53	9.5	10.38	0.91
	2016SHL	89.8	87.2	76-148.3	95.97	10.49	10.93	
	2017SHF	97.67	96.33	81.33-143	103.3	10.65	10.31	
	2017SHL	99	98.8	66.63-121.2	91.39	9.73	10.65	
	2018SHF	91.7	87.08	65.08-131.9	93.9	11.82	12.59	
	2018SHL	94.61	90	70.8-135.4	95.57	11.32	11.84	
	2019SHF	90.05	85.9	69.45-126.8	98.74	9.89	10.02	
	2019SHL	93.33	89.3	78.5-127.4	97.58	8.98	9.21	
	BLUP	93.24	91.91	74.65-127.5	96	9.14	9.52	
GNS	2017SHF	54	52	24-81.2	51.01	10.39	20.38	0.66
	2017SHL	44.5	43.6	26-77	41.94	8.08	19.27	
	2018SHF	54.6	49.9	31.6-70.8	45.62	6.11	13.4	
	2018SHL	54.5	54.1	35.3-70.8	52.07	7.18	13.78	
	2019SHF	55.7	53.7	35.2-84.6	53.66	8.18	15.24	
	2019SHL	56.5	56.2	35.5-75.8	53.77	7.07	13.15	
	BLUP	53.17	52.44	37.76-66.18	49.85	4.62	9.26	
SL	2016SHF	12.18	9.96	8.67-18	13.09	1.75	13.37	0.88
	2016SHL	12.1	9	6.65-14	10.53	1.61	15.33	
	2017SHF	13.5	11.5	8.5-17.88	13.04	1.73	13.23	
	2017SHL	13	11.5	8.33-17.67	12.93	1.88	14.51	
	2018SHF	11.85	9.26	7.63-14.93	11.82	1.84	15.54	
	2018SHL	13.02	10.9	7.55-15.7	11.3	1.72	15.18	
	2019SHF	13.71	11.2	8.89-18.17	13.25	1.87	14.15	
	2019SHL	12.4	10.5	8.5-16.3	12.51	1.56	12.51	
	BLUP	12.71	11.6	8.45-15.69	12.31	1.5	12.22	
SNS	2017SHF	18.6	19.6	16.2-25	19.58	1.39	7.08	0.4
	2017SHL	21.2	21.2	18-27	21.4	1.63	7.63	
	2018SHF	21.9	21.5	17.7-24.5	21.66	1.13	5.2	
	2018SHL	20.9	20.7	17.9-25.2	21.02	1.22	5.81	
	2019SHF	21.7	21.2	17.9-25	21.29	1.2	5.63	
	2019SHL	17.2	18.1	15.83-21.2	18.35	1.04	5.67	
	BLUP	20.3	20.35	18.42-22.96	20.55	0.84	4.1	

*SHF* Shifang, *SHL* Shuangliu, *BLUP* best linear unbiased prediction, *CV* coefficient of variation, *H*^*2*^ broad-sense heritability

### Correlation analyses among different traits

The BLUP datasets of each trait was employed to assess their correlations in the CM42×CM39 RIL population. TGW had significantly positive correlation with GW, GL, PH and SL, and significantly negative correlation with GNS and SNS (*P* < 0.001) (Fig. 1). GW was significantly and positively correlated with GL (*P* < 0.001), weakly and positively correlated with SL (*P* < 0.05), significantly and negatively correlated with GNS and SNS (*P* < 0.001), and not correlated with PH, respectively (Fig. 1). GL had significantly positive correlation with PH and SL (*P* < 0.001), significantly negative correlation with GNS (*P* < 0.001), and weakly negative correlation with SNS (*P* < 0.05) (Fig. 1). Significantly positive correlations between PH and SL, GNS and SNS, and SL and SNS (*P* < 0.001), weakly positive correlations between PH and SNS (*P* < 0.05), significantly negative correlations between PH and GNS (*P* < 0.001), and no correlations between GNS and SL were detected, respectively (Fig. 1). Grain weight per spike (GWS) is comprised by TGW and GNS in wheat. Thus, we further analyzed the correlation between the seven yield-related traits and the GWS. The results showed that GWS was significantly positive and positively correlated with TGW, GW, GL, GNS, SNS and SL (*P* < 0.05), and no correlated with PH (Table S4).

### QTL detection

Phenotypic data of the seven yield-related traits in each environment and the BLUP datasets were used for QTL detection, in which the BLUP datasets were treated as an additional environment. A total of 30 QTLs were identified in multi-environments and located on all chromosomes excepting 3B, 3D, 4B, 4D, 5D and 6B (Table 2).

**Table 2:** Quantitative trait loci (QTLs) for thousand grain weight (TGW), grain number per spike (GNS), grain width (GW), grain length (GL), plant height (PH), spike length (SL) and spikelet number per spike (SNS) identified across multi-environments in the CM42×CM39 RIL population

Trait	QTL	Env.	Chr.	Interval (cM)
TGW	*QTgw.cib-6A.1*	18SHF/18SHL/BLUP	6A	41.3-42.46
	*QTgw.cib-6A.2*	17SHF/18SHL/19SHF/19SHL/BLUP	6A	52.98-59.52
GW	*QGw.cib-2A*	18SHF/19SHF	2A	14.86-17.08
	*QGw.cib-2B.1*	19SHF/19SHL	2B	39.6-43.07
	*QGw.cib-2B.2*	17SHF/BLUP	2B	121.67-121.93
	*QGw.cib-5A*	17SHF/BLUP	5A	27.76-27.97
	*QGw.cib-6A*	17SHF/17SHL/18SHL/19SHF/19SHL/BLUP	6A	49.98-58.87
	*QGw.cib-7B*	18SHF/19SHL	7B	179.93-180.13
GL	*QGl.cib-3A*	17SHF/17SHL/18SHL/19SHF/19SHL/BLUP	3A	64.7-66.41
	*QGl.cib-5A.1*	17SHL/BLUP	5A	3.46-7.55
	*QGl.cib-5A.2*	18SHL/19SHF	5A	86.87-87.49
	*QGl.cib-6A*	17SHF/18SHF/18SHL/19SHF/19SHL/BLUP	6A	42.36-43.4
	*QGl.cib-6D*	18SHL/19SHF/BLUP	6D	76.06-83.69
	*QGl.cib-7D*	17SHF/17SHL/BLUP	7D	32.68-38.76
PH	*QPh.cib-1A*	16SHF/17SHL/19SHF	1A	28.34-30.95
	*QPh.cib-2D*	16SHF/17SHL/18SHF/18SHL/19SHF/BLUP	2D	1.48-5.16
	*QPh.cib-4A*	16SHF/17SHL	4A	82.78-83.05
	*QPh.cib-5A*	17SHF/19SHL/BLUP	5A	126.27-126.52
	*QPh.cib-5B*	16SHF/17SHL	5B	134.43-134.74
	*QPh.cib-6A*	16SHF/17SHL/19SHF	6A	54.61-54.76
GNS	*QGns.cib-2D*	18SHF/19SHF/BLUP	2D	0-5.16
	*QGns.cib-6A*	18SHL/19SHF/BLUP	6A	56.45-59.52
SL	*QSl.cib-2D*	16SHF/16SHL/17SHF/17SHL/18SHF/18SHL/19SHF/19SHL/BLUP	2D	1.48-5.16
	*QSl.cib-5A*	18SHL/19SHF/BLUP	5A	17.71-21.48
	*QSl.cib-5B*	16SHF/16SHL/17SHF/BLUP	5B	40.07-40.38
	*QSl.cib-6A*	19SHF/BLUP	6A	58.87-64.37
SNS	*QSns.cib-1B*	17SHF/18SHL/19SHF/BLUP	1B	28.8-33.3
	*QSns.cib-1D*	17SHL/BLUP	1D	146.67-148.82
	*QSns.cib-4A*	17SHF/18SHL/19SHF/BLUP	4A	72.98-81.71
	*QSns.cib-7A*	19SHF/19SHL	7A	81.76-85.42

Trait	Flanking Markers	LOD	PVE(%)	Add
TGW	Marker87546-Marker87736	6.17/8.03/4.44	13.49/16.38/9.89	-1.89/-2/-1.04
	Marker90290-Marker91587	9.48/7.95/7.27/10.52/9.62	20.39/16.68/15.31/20.51/23.75	-2.58/-2.06/-2.36/-2.88/-1.65
GW	Marker26336-Marker26958	5.48/2.76	9.51/5.81	0.05/0.04
	Marker29502-Marker29525	2.66/4.09	5.46/6.62	-0.04/-0.05
	Marker34419-Marker34417	3.83/3.7	5.2/5.2	-0.04/-0.03
	Marker70243-Marker70216	3.91/4.73	5.29/6.72	0.04/0.03
	Marker90210-Marker91133	13.09/4.95/8.93/4.02/5.8/14.53	19.87/8.92/19.17/8.6/10.1/23.31	-0.08/-0.09/-0.07/-0.05/-0.06/-0.06
	Marker111000-Marker110965	5.36/5.98	9.07/9.89	-0.05/-0.06
GL	Marker40793-Marker40901	5.31/2.97/6.1/5.69/3.87/5.68	11.86/6.55/10.17/10.31/7.37/9.54	-0.13/-0.08/-0.12/-0.1/-0.09/-0.09
	Marker69377-Marker69395	2.83/3.66	6.28/6.26	-0.08/-0.07
	Marker71923-Marker71919	3.79/3.82	6.13/6.76	-0.09/-0.08
	Marker87807-Marker87738	5.32/4.62/7.72/3.39/5.37/7.41	11.85/10.15/13.11/5.96/10.37/12.7	-0.13/-0.12/-0.13/-0.08/-0.11/-0.1
	Marker99119-Marker99140	4.17/5/3.17	6.95/8.98/5.17	0.1/0.1/0.06
	Marker111521-Marker111597	3/4.64/6.19	6.63/11.34/10.49	-0.09/-0.11/-0.09
PH	Marker5758-Marker6328	5.58/6.07/3.64	7.62/7.53/5.87	2.96/3.03/2.65
	Marker35344-Marker35422	3.42/4.31/3/4.7/3.14/2.56	4.54/5.23/6.73/9.38/5.03/6.2	2.29/2.53/3/3.72/2.46/2.31
	Marker57956-Marker57959	4.76/5.71	6.43/7.05	-2.73/-2.95
	Marker72631-Marker72950	3.02/2.91/2.52	7.18/7.03/5.62	-2.8/-2.32/-2.2
	Marker83905-Marker83879	3.07/3.18	4.07/3.8	2.17/2.16
	Marker90459-Marker90388	6.13/8.86/5.6	8.42/11.37/9.25	-3.24/-3.88/-3.46
GNS	Marker35164-Marker35422	2.57/2.63/4.73	5.73/4.97/6.46	-1.44/-1.93/-1.27
	Marker90628-Marker91587	3.35/3.83/4.91	7.73/7.46/6.56	2.07/2.46/1.33
SL	Marker35344-Marker35422	3.42/6.86/6.82/8.05/8.05/8.83/4.82/4.43/7.43	6.84/11.15/9.46/10.91/14.89/13.41/6.18/8.31/13.51	0.46/0.6/0.66/0.76/0.75/0.7/0.53/0.48/0.58
	Marker69427-Marker69525	2.59/6/2.61	3.47/7.8/4.22	-0.36/-0.6/-0.32
	Marker81580-Marker81513	2.99/3.06/2.87/2.51	5.96/4.76/3.79/4.04	0.43/0.39/0.42/0.32
	Marker91133-Marker91933	4.62/3	5.9/5.63	-0.54/-0.39
SNS	Marker15740-Marker17413	16.86/4.13/6.11/5.4	16.18/7.47/9.85/8.21	0.82/0.35/0.4/0.25
	Marker23471-Marker23475	5.65/4.49	8.39/6.77	-0.5/-0.22
	Marker57882-Marker57915	2.51/4.94/5.3/5.63	2.34/10.46/9.69/9.68	-0.31/-0.41/-0.39/-0.27
	Marker103527-Marker103903	3.25/4.47	5.06/8.18	0.28/0.3

*PVE* mean of phenotypic variation explained, *LOD* logarithm of the odd, *Add* additive effect (Positive values indicate that the alleles from CM39 increases the trait scores, and negative values indicate that the allele from CM42 increases the trait scores), *BLUP* best linear unbiased prediction, *Chr.* chromosome, *Env.* environment

For TGW, two QTLs were detected on chromosomes 6A. *QTgw.cib-6A.1* was detected in two environments and the BLUP datasets, explaining 9.89-16.38% of the phenotypic variance. *QTgw.cib-6A.2* was a major QTL detected in four environments and the BLUP datasets and explained 15.31-23.75% of the phenotypic variance. Alleles of CM42 for the two QTLs contributed to higher TGW (Table 2).

For GW, six QTLs were identified on chromosomes 2A, 2B, 5A, 6A and 7B. Of them, a major QTL *QGw.cib-6A* was identified in five environments and the BLUP datasets, explaining 8.6-23.31% of the GW variation. The allele of CM42 contributed positively to the GW. The rest five minor QTLs were identified in two environments and explained 5.2-9.89% of the GW variation. The favorable alleles of *QGw.cib-2A* and *QGw.cib-5A* were contributed by CM39, and that of *QGw.cib-2B.1*, *QGw.cib-2B.2* and *QGw.cib-7B* were contributed by CM42 (Table 2).

Among the six QTLs for GL, two major QTL *QGl.cib-3A* and *QGl.cib-6A* were identified in five environments and the BLUP datasets, explaining 6.55-11.86% and 5.96-13.11% of the GL variation, respectively. The positive additive effects of the two QTLs on GL were contributed by CM42. The rest four minor QTLs were identified in two or three environments on chromosome 5A, 6D and 7D, explaining 5.17-11.34% of the GL variation. The favorable alleles of *QGl.cib-5A.1*, *QGl.cib-5A.2*, and *QGl.cib-7D* were derived from CM42, and that of *QGl.cib-6D* was derived from CM39 (Table 2).

Among the six QTLs for PH, *QPh.cib-2D* on chromosome 2D was a stable QTL and detected in five environments and the BLUP datasets, explaining 4.54-9.38% of the PH variation. The allele of CM39 contributed to higher PH. The rest five minor QTLs on chromosomes 1A, 4A, 5A, 5B and 6A were detected in two or three environments, explaining 3.8-11.37% of the PH variation. The positive alleles of *QPh.cib-1A* and *QPh.cib-5B* were from CM39, and that of *QPh.cib-4A*, *QPh.cib-5A* and *QPh.cib-6A* were from CM42 (Table 2).

Two minor QTLs for GNS on chromosomes 2D and 6A were detected in two environments and the BLUP datasets and explained 4.97-6.46% and 6.56-7.73% of the GNS variation, respectively. Alleles from CM42 and CM39 at *QGns.cib-2D* and *QGns.cib-6A*, respectively, contributed to positive effects on GNS (Table 2).

For SL, four QTLs were detected on chromosomes 2D, 5A, 5B and 6A. A major QTL *QSl.cib-2D* was detected in eight environments and the BLUP datasets, explaining 6.18-14.89% of the SL variation. *QSl.cib-5B* was a stable QTL and detected in three environments and the BLUP datasets, explaining 3.79-5.96% of the SL variation. Alleles of CM39 for the two QTLs contributed to increase of SL. Two minor QTLs *QSl.cib-5A* and *QSl.cib-6A* were detected in two or three environments, explaining 3.47-7.8% and 5.63-5.9% of the SL variation, respectively. The positive alleles of the two QTLs were contributed by CM42 (Table 2).

Four QTLs for SNS were identified on chromosomes 1B, 1D, 4A and 7A. Of them, *QSns.cib-1B* and *QSns.cib-4A* were detected in three environments and the BLUP datasets, explaining 7.47-16.18% and 2.34-10.46% of the SNS variation, respectively. *QSns.cib-1D* and *QSns.cib-7A* were detected in two environments, explaining 6.77-8.39% and 5.06-8.18% of the SNS variation, respectively. The favorable alleles of *QSns.cib-1B* and *QSns.cib-7A* were contributed by CM39, and that of *QSns.cib-1D* and *QSns.cib-4A* were contributed by CM42 (Table 2).

### Effects of major QTL in mapping populations

**Fig. 2:**
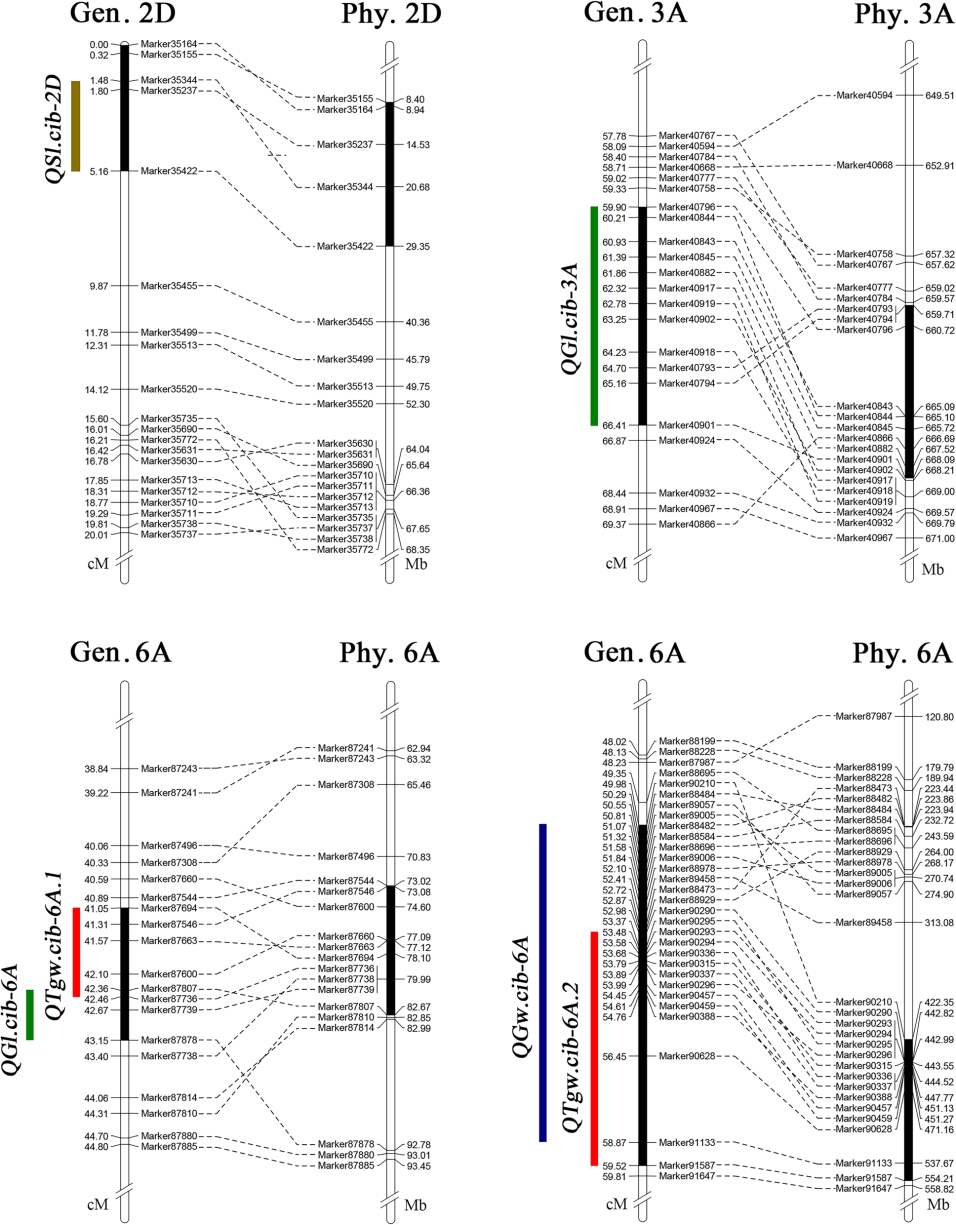
The genetic and physical position of six major QTLs*, QSl.cib-2D*, *QGl.cib-3A*, *QTgw.cib-6A.1*, *QTgw.cib-6A.2*, *QGw.cib-6A*, and *QGl.cib-6A* detected in the CM42 ×CM39 RIL population; *Chr.*, genetic position; *Phy.*, physical position

**Fig. 3:**
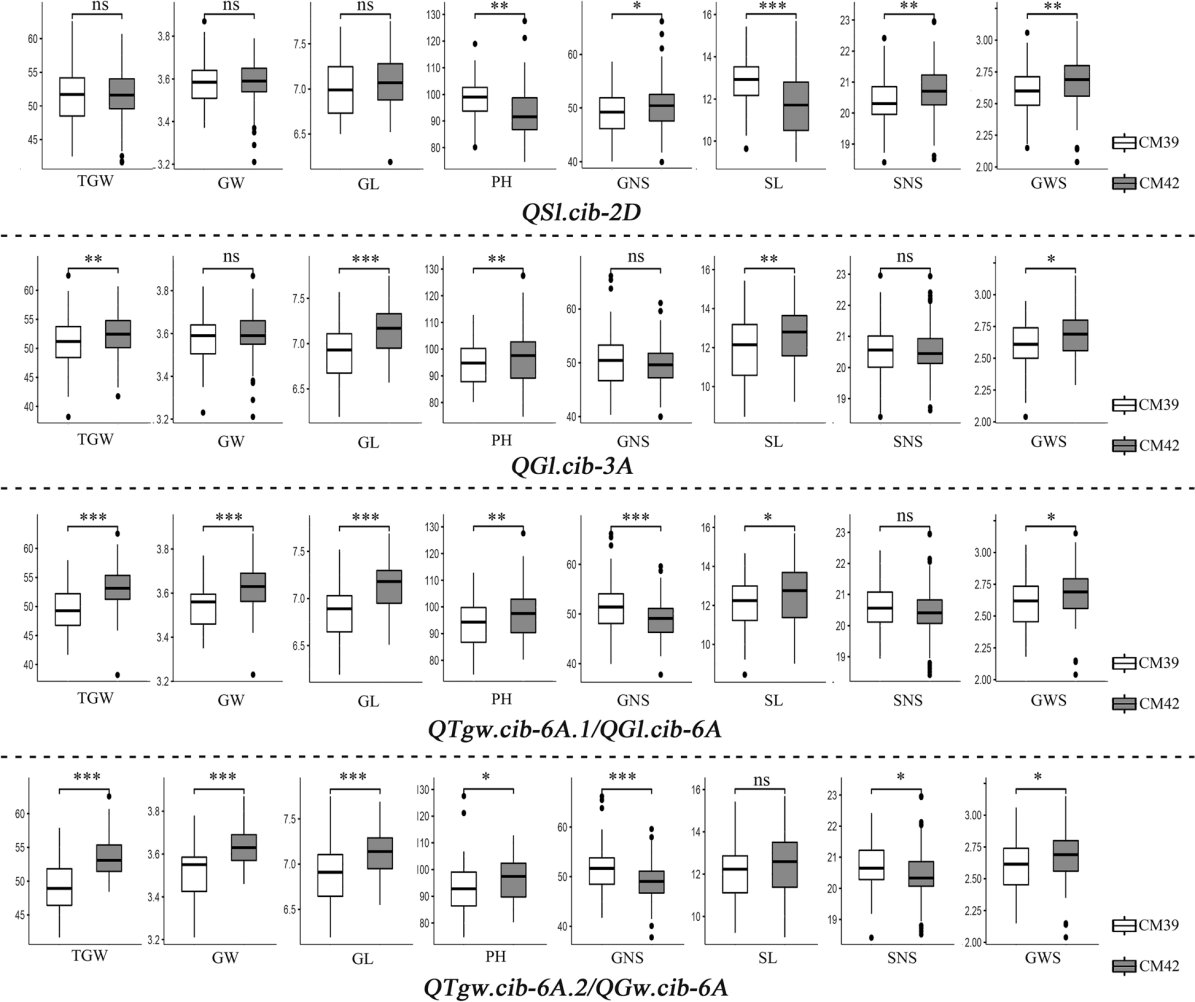
Effects of major QTLs, *QSl.cib-2D*, *QGl.cib-3A*, *QTgw.cib-6A.1*, *QGl.cib-6A*, *QTgw.cib-6A.2*, and *QGw.cib-6A*, on seven yield-related traits and grain weight per spike (GWS) in the CM42×CM39 RIL population. *CM42* and *CM39* indicate the lines with the alleles from CM42 and CM39, respectively; *, ** and *** represent significance at *P* < 0.05, *P* < 0.01, and *P* < 0.001, respectively; *ns* represents non-significance

Six major QTLs *QSl.cib-2D*, *QGl.cib-3A*, *QTgw.cib-6A.1*, *QTgw.cib-6A.2*, *QGw.cib-6A*, and *QGl.cib-6A* were stably identified in multi-environments and the BLUP datasets (Table 2, Fig. 2). Based on the physical position of the flanking markers of them, three Kompetitive Allele Specific PCR (KASP) markers, *K_2D-20925377*, *K_6A-83647812*, and *K_6A-54337781*, tightly linked to *QSl.cib-2D, QTgw.cib-6A.1/QGl.cib-6A*, and *QTgw.cib-6A.2/QGw.cib-6A*, respectively, were successfully developed (Table S5, Fig. S1). We further analyzed the effects of these major QTLs on the seven yield-related trait and GWS using the three KASP markers and the flanking markers of *QGl.cib-3A* in the CM42×CM39 RIL population. The results showed that *QSl.cib-2D* significantly affected PH, GNS, SL, SNS and GWS, *QGl.cib-3A* significantly affected TGW, GL, PH, SL and GWS, *QTgw.cib-6A.1/QGl.cib-6A* significantly affected TGW, GW, GL, PH, GNS, SL and GWS, and *QTgw.cib-6A.2*/*QGw.cib-6A* significantly affected TGW, GW, GL, PH, GNS, SNS and GWS (Fig. 3).

### QTL clusters on chromosome 2D and 6A

The QTL cluster on 2D, including three QTLs *QSl.cib-2D*, *QPh.cib-2D* and *QGns.cib-2D*, was co-located between Marker35164 and Marker35422 (Table 2). Two QTL clusters were identified on chromosome 6A. One comprised two QTLs, *QTgw.cib-6A.1* and *QGl.cib-6A*, was located between Marker87546 and Marker87738 (Table 2). The other one contained five QTLs, *QTgw.cib-6A.2*, *QGw.cib-6A*, *QPh.cib-6A*, *QGns.cib-6A* and *QSl.cib-6A*, was located between Marker90210 and Marker91587 (Table 2).

## Discussion

### QTL analysis and comparison with previous studies

Wheat yield-related traits are significantly associated with yield and typically show higher heritability than the yield itself, and thus, mining the genes or QTLs related to yield-related traits will be help for elucidating the genetic basis of wheat yield and facilitating the genetic improvement of varieties with high yield [5–7]. In the present study, a RIL population derived from two elite winter wheat varieties were used to dissect the genetic basis of variation for seven yield-related traits, including TGW, GNS, GW, GL, PH, SL and SNS. A total of 30 QTLs were identified in multiple environments, explaining 2.34-23.75% of the phenotypic variance (Table 2).

Fourteen QTLs were identified for grain size and weight, including two for TGW, six for GW and six for GL. Among them, *QTgw.cib-6A.1* and *QGl.cib-6A* were co-located on chromosome arm 6AS, which was near to *QTkw-6A.1* and *QTgw.cau-6A.4* [1, 43]. *QTgw.cib-6A.2* was located on chromosome arm 6AL and near to *QTKW.caas-6AL* and *QTKW-6A.1* [44, 45]. The QTL *QGw.cib-6A* for GW was located in a large interval on chromosome 6A. This interval was near to a known gene *TaGW2* controlling TGW and GW [46, 47]. *QGw.cib-2A* on chromosome 2A was overlapped with *QGwt.crc-2A* detected by McCartney et al [48]. *QGw.cib-2B.1* on chromosome 2B was overlapped with *qKW2B-1* detected by Xin et al [30]. *QGw.cib-7B* on chromosome 7B was located near to a QTL for TGW *QTgw.wa-7BL* [6]. Two QTLs for GL *QGl.cib-3A* and *QGl.cib-5A.1* on chromosomes 3A and 5A, respectively, were overlapped with two QTLs for GL detected by Mohler et al [49]. *QGl.cib-5A.2* was near to a QTL for TGW *QTKW.ndsu.5A.1* reported previously [47]. *QGl.cib-7D* was overlapped with *QGl.cau-7D* detected by Yan et al [50]. For the rest three QTLs *QGw.cib-2B.2*, *QGw.cib-5A* and *QGl.cib-6D*, no stable QTL for grain size reported previously was overlapped with them, indicating they are likely novel QTL (Table 3).

**Table 3:** The physical interval of QTL detected in the present study and comparison with previously studies.

Trait	QTL	Chromosome	Physical position (Mb)	Nearby known locus	Reference
TGW	*QTgw.cib-6A.1*	6A	73.08-82.67	*QTkw-6A.1, QTgw.cau-6A.4*	[1, 43]
	*QTgw.cib-6A.2*	6A	442.82-554.21	*QTKW.caas-6AL, QTKW-6A.1*	[44, 45]
GW	*QGw.cib-2A*	2A	517.02-581.44	*QGwt.crc-2A*	[48]
	*QGw.cib-2B.1*	2B	150.75-151.74	*qKW2B-1*	[30]
	*QGw.cib-2B.2*	2B	734.72-734.72		
	*QGw.cib-5A*	5A	202.92-212.92		
	*QGw.cib-6A*	6A	422.35-537.67	*TaGW2*	[46, 47]
	*QGw.cib-7B*	7B	735.93-740.06	*QTgw.wa-7BL*	[6]
GL	*QGl.cib-3A*	3A	659.71-668.09	*IWA4298-IWB11347*	[49]
	*QGl.cib-5A.1*	5A	26.14-29.28	*IWA4871-IWB34408*	[49]
	*QGl.cib-5A.2*	5A	453.5-453.6	*QTKW.ndsu.5A.1*	[47]
	*QGl.cib-6A*	6A	79.99-82.67		
	*QGl.cib-6D*	6D	75.08-83.92		
	*QGl.cib-7D*	7D	66.19-107.61	*QGl.cau-7D*	[50]
PH	*QPh.cib-1A*	1A	345.37-443.28		
	*QPh.cib-2D*	2D	20.68-29.35	*Rht8, QPLH-2D*	[31, 51]
	*QPh.cib-4A*	4A	704.53-704.58	*Chr4A-B57-Hap6*	[52]
	*QPh.cib-5A*	5A	501.62-523.22	*Chr5A-B54-Hap3*	[52]
	*QPh.cib-5B*	5B	607.07-608.06		
	*QPh.cib-6A*	6A	447.77-451.27	*Rht18*	[53]
GNS	*QGns.cib-2D*	2D	8.4-29.35	*Rht8*	[31, 51]
	*QGns.cib-6A*	6A	471.16-554.21	*QTKW.caas-6AL, QTKW-6A.1*	[44, 45]
SL	*QSl.cib-2D*	2D	20.68-29.35	*Rht8, QPLH-2D*	[31, 51]
	*QSl.cib-5A*	5A	35.84-45.91	*QSL5A.3*	[54]
	*QSl.cib-5B*	5B	404.42-406.31		
	*QSl.cib-6A*	6A	537.67-584		
SNS	*QSns.cib-1B*	1B	381.92-439.8	*QSn.sau-1BL*	[5]
	*QSns.cib-1D*	1D	482.32-485.76		
	*QSns.cib-4A*	4A	691.53-703.17		
	*QSns.cib-7A*	7A	524.95-562.63	*QSn-7A.2*	[55]

PH and SL are important traits related to plant architecture and yield potential in wheat [12, 56]. In the present study, six and four QTLs for PH and SL were identified, respectively. Among them, *QPh.cib-2D* and *QSl.cib-2D* were co-located in the same interval on chromosome arm 2DS, which was overlapped with the dwarfing gene *Rht8* [31, 51]. *QPh.cib-4A* and *QPh.cib-5A* were located near to two loci for PH reported by Luján Basile et al [52]. *QPh.cib-6A* on chromosome 6A was overlapped with the dwarfing gene *Rht18* [53]. *QSl.cib-5A* on chromosome 5A was located near to *QSL5A.3* detected by Liu et al [54]. For the rest four QTLs *QPh.cib-1*A, *QPh.cib-5B*, *QSl.cib-5B* and *QSl.cib-6A*, no stable QTL for PH and SL reported previously was overlapped with them, indicating they are likely novel (Table 3).

Two QTLs for GNS and four QTLs for SNS were identified in the present study. Of them, *QGns.cib-2D* were co-located with *QPh.cib-2D* and *QSl.cib-2D* on chromosome 2D and overlapped with the dwarfing gene *Rht8* [31, 51]. *QGns.cib-6A* was co-located with *QTgw.cib-6A.2* and near to two QTLs for TGW *QTKW.caas-6AL* and *QTKW-6A.1* [44, 45]. *QSns.cib-1B* for SNS on chromosome 1B was overlapped with the *QSn.sau-1BL* reported recently [5]. *QSns.cib-7A* for SNS on chromosome 7A was overlapped with *QSn-7A.2* detected by Cao et al [55]. For the rest two QTLs *QSns.cib-1D* and *QSns.cib-4A*, no stable QTL for SNS reported previously was overlapped with them, indicating they are likely novel (Table 3).

### QTL cluster on chromosomes 2D and 6A

Numerous co-located QTLs associated with multiple traits have been reported in the previous studies [2, 5, 24, 57, 58], which are beneficial to improve breeding efficiency for multiple elite traits, and thus is favorable for pyramiding breeding. In the present study, three QTLs *QSl.cib-2D*, *QPh.cib-2D* and *QGns.cib-2D* were co-located in the interval of 8.4-29.35 Mb on chromosome arm 2DS (Table 2). The allele of CM42 at the locus decreases SL and PH while increasing GNS. Additionally, the locus was overlapped with the dwarfing gene *Rht8*, which has been reported to associated with QTLs for PH, SL, SNS, GNS, spikelet compactness, TGW, and grain yield [12, 51, 59–61]. Interestingly, no QTL for grain size and weight detected in the present study was overlapped with the locus, indicating it had no effect on grain size and weight. Given CM42 was bred by utilizing synthetic wheat germplasm [62], further studies, such as fine-mapping and map-based cloning are needed to future reveal the relationship between the locus and *Rht8*. However, the results in this study showed that the locus could be utilized in optimization PH with no penalty for grain size and weight in MAS.

Two QTL clusters were detected on chromosome 6A in the present study. One comprised two QTLs, *QTgw.cib-6A.1* and *QGl.cib-6A,* was located on chromosome arm 6AS (Table 2, Fig. 2). The other one comprised five QTLs, *QTgw.cib-6A.2*, *QGw.cib-6A*, *QGns.cib-6A*, *QPh.cib-6A*, and *QSl.cib-6A*, was located on chromosome arm 6AL (Table 2, Fig. 2). The QTL cluster on chromosome 6AL was overlapped with the haplotype block encompassing *TaGW2* and additional 2167 genes which was located between 187 Mb and 455 Mb on chromosome 6A and defined by Brinton et al [63]. Therefore, fine-mapping and map-based cloning is needed to dissect the relationships between *TaGW2* and the QTL cluster on chromosome 6AL in the future study. For the QTL cluster on chromosome 6AS, which was located between 73.08 Mb and 82.67 Mb and far apart the haplotype block of *TaGW2* [63], indicating that they are different loci for grain weight.

### Additive effects of three major QTLs on TGW and GNS

Due to there is a trade-off between TGW and GNS, increasing one of them may not contribute to an increase in grain yield of wheat. We further analyzed the additive effects of three major QTLs, *QPh/Sl.cib-2D*, *QGl.cib-3A* and *QTgw.cib-6A.2*, on the TGW and GNS. As showed in the Table 4, lines possessing the allele from CM42 at the three loci had relatively higher TGW and GNS, which might partly explain the high yield of CM42. Additionally, lines possessing the alleles from CM42 at *QPh/Sl.cib-2D* and *QTgw.cib-6A.2* and the allele from CM39 at *QGl.cib-3A* also had relatively higher TGW and GNS. However, for the other combination schemes, either the higher TGW but lower GNS, or higher GNS but lower TGW, or both lower TGW and GNS were harvested. Overall, the QTLs and KASP markers in this study will be useful for elucidating the genetic architecture of grain yield and developing new wheat varieties with high and stable yield in wheat.

**Table 4:** Analyses of additive effects on TGW and GNS of three major QTLs *QPh/Sl.cib-2D*, *QGl.cib-3A* and *QTgw.cib-6A.2*

QTL	Lines	TGW(g) ^**^	GNS ^*^
aabbcc	20	46.97±2.85 ^a^	52.25±5.25 ^cd^
AABBcc	14	47.63±3.29 ^a^	49.78±5.12 ^abc^
AAbbcc	7	49.75±3.76 ^ab^	55.2±5.51 ^d^
aaBBcc	16	51.53±2.42 ^bc^	50.58±3.48 ^bc^
aabbCC	20	53.13±3.49 ^cd^	47.74±4.1 ^a^
AAbbCC	18	53.17±2.17 ^cd^	50.58±4 ^bc^
aaBBCC	21	54.25±2.94 ^d^	48.84±3.47 ^ab^
AABBCC	31	53.32±2.88 ^d^	50.07±2.86 ^bc^

*aa*, *bb* and *cc* represent the allele from CM39 at *QPh/Sl.cib-2D*, *QGl.cib-3A* and *QTgw.cib-6A.2*, respectively; *AA*, *BB* and *CC* represent the allele from CM42 at *QPh/Sl.cib-2D*, *QGl.cib-3A* and *QTgw.cib-6A.2*, respectively; *Lines* represent the number of different haplotypes; ^*^ and ^**^ represent significance at *P* < 0.05 and *P* < 0.01, respectively; The superscript letter indicates significant difference among groups

### Potential candidate genes for *QTgw.cib-6A.1/QGl.cib-6A* and *QGl.cib-3A*

Among these major QTL, *QSl.cib-2D* is likely allele with *Rht8*. In the previous study, *TraesCS2D01G055700* was reported by Chai et al [64] as a possible candidate gene of *Rht8*. *QTgw.cib-6A.2*/*QGw.cib-6A* was needed additional populations to narrow their physical interval. Therefore, we mainly analyzed possible candidates for *QTgw.cib-6A.1/QGl.cib-6A* and *QGl.cib-3A* in the present study.

**Fig. 4:**
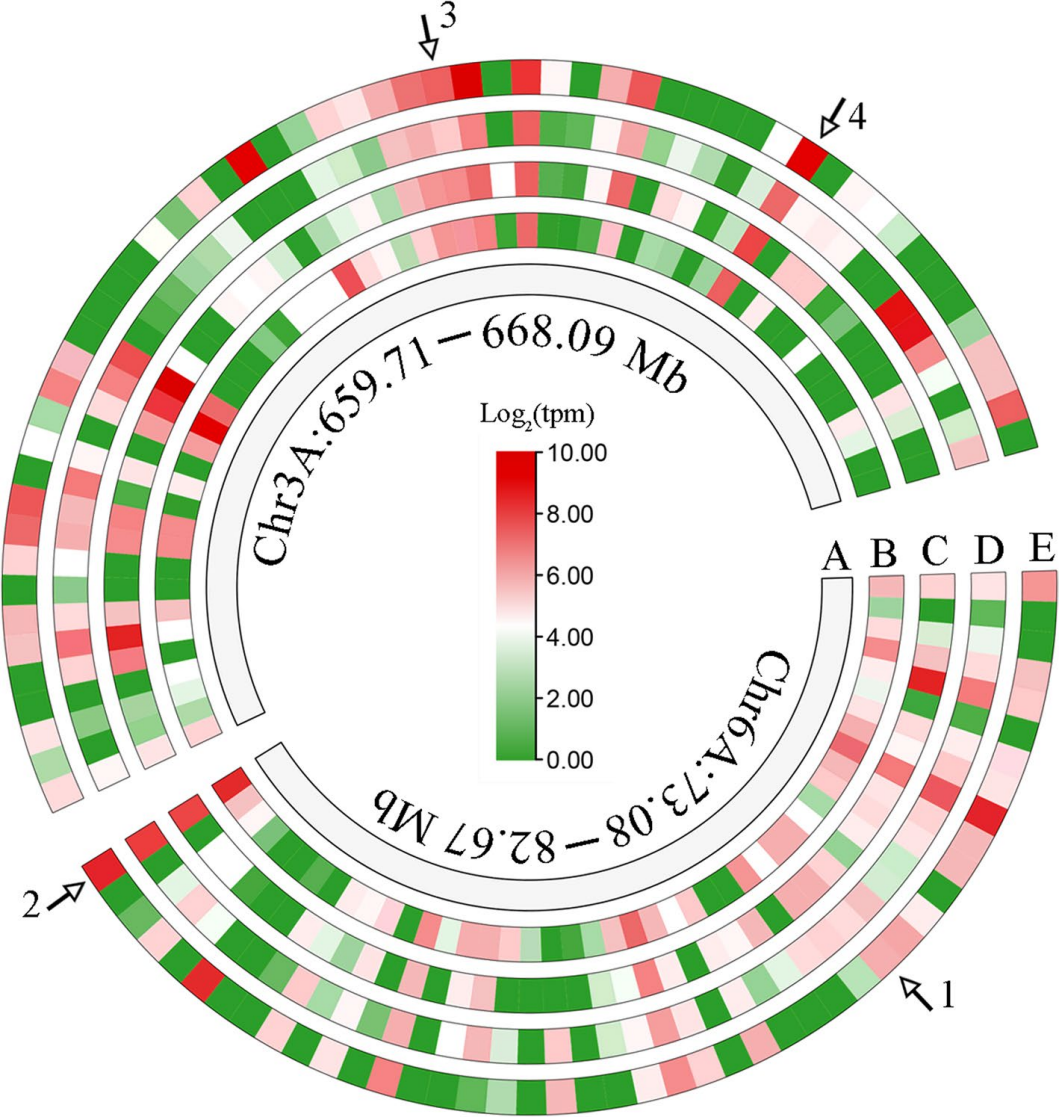
Expression pattern of genes within the *QTgw.cib-6A.1/QGl.cib-6A* and *QGl.cib-3A* intervals. *1*, *2*, *3* and *4* marked by the arrow represent *TraesCS6A02G107800*, *TraesCS6A02G112400*, *TraesCS3A02G421900* and *TraesCS3A02G424000*, respectively; *A* represents the physical interval of *QTgw.cib-6A.1/QGl.cib-6A* and *QGl.cib-3A* on chromosome 6A and 3A; *B*, *C*, *D* and *E* represent root, leaf/shoot, spike and, grain, respectively

*QTgw.cib-6A.1* and *QGl.cib-6A* were co-located between 73.08 and 82.67 Mb on Chinese Spring (CS) chromosome arm 6AS, and *QGl.cib-3A* was located between 659.71 and 668.09 Mb on CS chromosome arm 3AL (Table 3, Fig. 2). In the interval of *QTgw.cib-6A.1/QGl.cib-6A* and *QGl.cib-3A*, there were 81 and 85 predicted genes in the CS genome, respectively (Table S6, S7). Expression pattern analyses showed that 45 and 57 genes in the interval of *QTgw.cib-6A.1/QGl.cib-6A* and *Gl.cib-3A* expressed in various tissue, respectively (Fig. 4) [65, 66]. Among them, several were abundantly expressed in grain, indicating they are likely associated with grain growth and development (Fig. 4). For example, *TraesCS6A02G107800* is an ortholog of the rice *RGG2* and encodes a guanine nucleotide-binding protein subunit gamma 2 (Table S6). Miao et al previously reported that *RGG2* played a negative role in plant growth and yield production and that manipulation of *RGG2* can increase the plant biomass, grain weight, length and yield in rice [67]. *TraesCS6A02G112400* and *TraesCS3A02G424000* encode polyubiquitin and small ubiquitin-related modifier, respectively (Table S6, S7). *TraesCS3A02G421900* encodes a 26S proteasome regulatory subunit (Table S7), which participates in the ubiquitin/26S proteasome pathway and mediate the degradation of the complex of ubiquitin receptor and poly-ubiquitinated protein [68, 69]. Previous studies revealed that the ubiquitin pathway play an important role in regulation grain size and weight in rice [70, 71]. These results indicated that the four genes may be closely related to grain size and weight in wheat and useful for fine mapping and cloning of *QTgw.cib-6A.1/QGl.cib-6A* and *QGl.cib-3A* in our following work.

## Conclusion

In this study, a total of 30 QTLs for TGW, GNS, GW, GL, PH, SL, and SNS were identified, explaining 2.34-23.75% of the phenotypic variance. Among them, six major QTLs *QTgw.cib-6A.1*, *QTgw.cib-6A.2*, *QGw.cib-6A*, *QGl.cib-3A*, *QGl.cib-6A*, and *QSl.cib-2D* were detected. Three KASP markers linked with five of these major QTLs were developed. These QTLs and KASP markers will be useful for elucidating the genetic architecture of grain yield and developing new wheat varieties with high and stable yield in wheat. Additionally, candidate genes of *QTgw.cib-6A.1/QGl.cib-6A* and *QGl.cib-3A* were preliminary analyzed.

## Methods

### Plant materials and field trials

A RIL population (F_10_) comprising 193 lines derived from a cross CM42 and CM39 were used for QTL detection in the present study. CM42 is the first wheat elite variety in the world bred by using synthetic hexaploid wheat (*Triticum turgidum×Aegilops tauschii*) germplasm, and showed high yield potential in Sichuan and the Yangzi River region [62], while CM39 is an elite winter wheat variety with different genetic background to that of CM42. During four growing seasons of wheat from 2015-2016 to 2018-2019, the RIL population along with their parents were evaluated at two experimental sites in Sichuan province of China, including Shuangliu (SHL, 103° 52'E, 30°34'N) and Shifang (SHF, 104°11'E, 31°6'N). Randomized block design was adopted for all of the trials. Each line was planted in a one-row plot with 50 seeds per row, a row length of 2.0 m, and a row spacing of 0.3 m. Five replicates were performed under each environment. Nitrogen and superphosphate fertilizers were applied at a rate of 80 and 100 kg/ha, respectively, at sowing. Crop management and disease control were performed according to local cultivation practices.

### Phenotyping and statistical analysis

At maturity, ten representative plants from middle row of each line were randomly selected to investigate agronomic traits including TGW, GL, GW, GNS, PH, SL, SNS and GWS. SL was measured as the length from the base of the rachis to the tip of the terminal spikelet, excluding the awns. SNS was determined by counting the number of spikelets in main spikes; PH was measured from the soil surface to the tip of the spike, excluding the awns. Subsequently, the main spike of all selected plants were harvested and manually threshed for evaluating GNS, TGW, GW, GL and GWS using SC-G software (Wseen Co., Ltd, Hangzhou, China). PH and SL were evaluated in eight environments, and the rest traits were evaluated in six environments.

Basic phenotypic statistical analyses, frequency distribution, correlation analyses and student’s t tests were performed with SPSS version 20.0 (Chicago, IL, USA). The phenotype distribution graph was drawn using the plugin “CorrPlot” in TBtools [72]. The relationships among measured traits were visualized using the R package “qgraph”. The BLUP data across evaluated environments was calculated using the “lmer” function implemented in R package “lme4”. ANOVA was performed over all trials which indicated statistically significant main effects for genotypes (G), environments (E), G × E interactions for all measured traits using the SAS software (SAS Institute Inc., North Carolina, USA). The broad sense heritability (*H*^*2*^) was estimated based on the following equation: ${H}^2={\sigma}_g^2/\left({\sigma}_g^2+{\sigma}_{ge}^2/n+{\sigma}_e^2/ nr\right)$, where${\sigma}_g^2$ is the variance of genotypes, ${\sigma}_{ge}^2$ is the variance of genotype by environmental effect, ${\sigma}_e^2$ is the residual variance, n is the number of environments and r is the number of replicates [73].

### Linkage map construction and QTL detection

A whole-genome genetic map constructed previously was adopted for QTL mapping [42]. The genetic map was constructed using the CM42×CM39 RIL population with SLAF markers. A total of 4996 Bin SLAFs were distributed in 21 linkage groups and covered a total genetic distance of 2,859.94 cM with an average interval of 0.57 cM between adjacent Bin marker (Table S1, S2) [42].

QTL analysis was conducted using the inclusive composite interval mapping (ICIM) function of IciMapping 4.1 (https://www.isbreeding.net) with the minimal LOD score was set at 2.5. The missing phenotype was deleted in QTL analysis. QTL was named according to the provision of Genetic Nomenclature (http://wheat.pw.usda.gov/ggpages/wgc/98/Intro.htm), where ‘CIB’ represents Chengdu Institute of Biology. QTLs consistently identified in at least three environments and in combined analysis with ≥10% of phenotypic variation explained were considered as major QTLs.

### Development of Kompetitive Allele-Specific PCR Markers

On the basis of the preliminary QTL mapping results, the flanking markers of major QTL were blasted against the CS reference genome sequence (RefSeq v1.0; https://wheat-urgi.versailles.inra.fr/) to gain their physical positions [74]. The SNPs within the physical interval of major QTLs were used for developing KASP markers tight linked with them. The KASP marker primers were designed using the PrimerServer tool in Triticeae Multi-omics Center (http://202.194.139.32/) [75]. Standard FAM and HEX adapters were added to the allele-specific forward primers at the 5′ ends. The KASP assays were run in a Bio-Rad CFX96 real-time PCR system in 10μL reaction volumes with the following PCR cycling parameters: hot start enzyme activation at 94 °C for 15 min; a touchdown of 10 cycles (94 °C for 20 s, and touchdown starting at 61 °C and decreasing by 0.6 °C per 1-min cycle); then 26 cycles of regular PCR (94 °C for 20 s, 55 °C for 60 s, and rest at 37 °C for 1 min). If the clustering was not significant, further cycling was performed at 94 °C for 20 s and 55 °C for 60 s (3–10 cycles per step)

### Prediction of candidate gene

Genes between the physical intervals of major QTLs were extracted from IWGSC RefSeq v1.1 annotation for CS [74]. The annotations and functions of a given gene were analyzed using UniProt (https://www.uniprot.org/). The expression pattern analysis was performed by using Wheat Expression Browser (http://www.wheat-expression.com/), and the circle graph of expression values was drawn using TBtools [72]. The orthologous gene analysis between wheat and rice was conducted using the Triticeae-Gene Tribe (http://wheat.cau.edu.cn/TGT/) [76].

## Supplementary Information

**Additional file 1.** 

**Additional file 2.** 

## Data Availability

All data used in this study was present in the manuscript and supporting materials.

## Abbreviations

CM42: Chuanmai42
CM39: Chuanmai39
SHF: Shifang
SHL: Shuangliu
TGW: Thousand grain weight
GNS: Grain number per spike
GW: Grain width
GL: Grain length
PH: Plant height
SL: Spike length
SNS: Spikelet number per spike
QTL: Quantitative trait loci
SLAF: Specific-locus amplified fragment
MAS: Marker-assisted selection
KASP: Kompetitive Allele Specific PCR
BLUP: Best linear unbiased prediction
CS: Chinese Spring
