# Supplementary figures and images for: High-resolution detection of quantitative trait loci for seven important yield-related traits in wheat (Triticum aestivum L.) using a high-density SLAF-seq genetic map
Source: BMC Genom Data. 2022 May 13;23:37. doi: 10.1186/s12863-022-01050-0 (PMC9107147; doi:10.1186/s12863-022-01050-0)

**
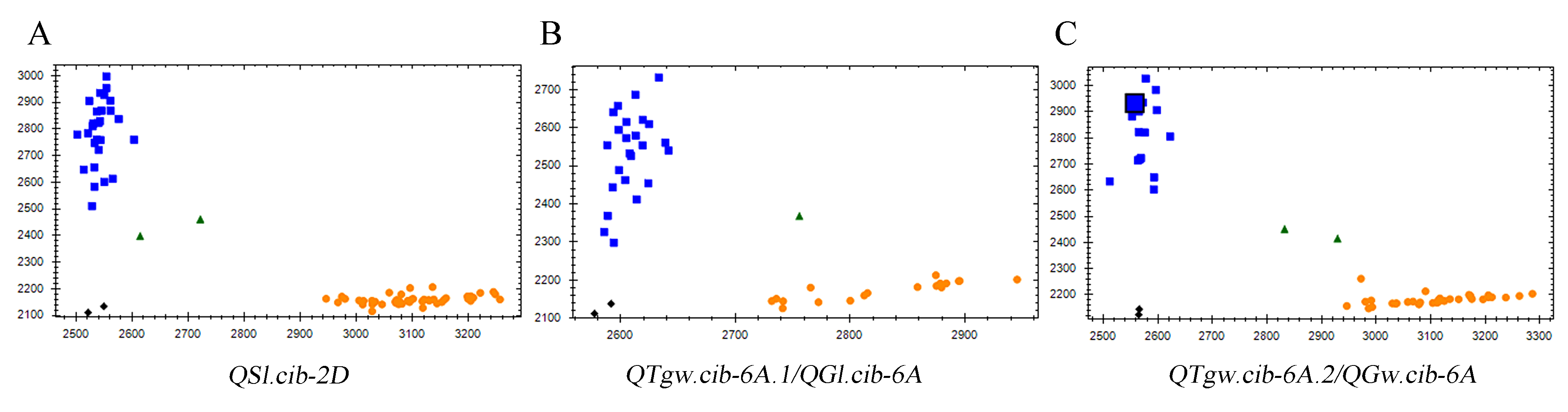
**

**Fig. S1** Genotyping of the CM42×CM39 RIL population using the three KASP marker, *K_2D-20925377* (**A**), *K_6A-83647812* (**B**), and *K_6A-54337781* (**C**).

Supplement: Supplementary file 1 — Additional file 1. [file 12863_2022_1050_MOESM1_ESM.docx]
